# Prevalence and Regulation of Dyslipidemia Among Adults With Type 2 Diabetes From Three Primary Health Care Centers in Riyadh

**DOI:** 10.7759/cureus.27573

**Published:** 2022-08-01

**Authors:** Fatemah S Bin Saleh, Weam S Alharbi, Ghadah B Alanazi, Aida Aldughaither

**Affiliations:** 1 Family Medicine, King Abdulaziz Medical City, Ministry of National Guard Health Affairs, Riyadh, SAU

**Keywords:** saudi arabia, prevalence, lipid profile, diabetes miletus, dyslipidemia

## Abstract

Background

Dyslipidemia is an added risk factor in patients with type 2 diabetes who are more prone to develop cardiovascular diseases as it implies an alteration of the lipid level leading to serious health complications.

Objective

This study aimed at assessing the prevalence of dyslipidemia among patients with type 2 diabetes and comparing the lipid profile measurements between controlled and uncontrolled type 2 diabetic patients.

Method

A cross-sectional retrospective study was performed in three primary health care centers in Saudi Arabia. A sample of 418 patients with type 2 diabetes was enrolled in this study. To collect data, the researcher used a structured questionnaire and retrieved patients' data from the electronic medical records in the study setting.

Results

The findings of the study showed that 82.1% of type 2 diabetes mellitus (DM) patients recruited in this study were dyslipidemic. In addition, it was found that there was a significant difference in triglycerides, fasting blood sugar (FBS), and high-density lipoprotein (HDL) between controlled and uncontrolled diabetic patients (p≤0.05). Moreover, a significant interaction was found between gender, HbA1c control, educational level, and frequency of exercising on one hand and dyslipidemia on the other hand (p≤0.05).

Conclusion

The study concluded that there is a high prevalence of dyslipidemia among type 2 diabetic patients in Saudi Arabia and a significant interaction between dyslipidemia and diabetic patients' gender, HbA1c control, educational level, and frequency of exercising. The study recommends increasing type 2 diabetic patients' awareness regarding the management of diabetes and dyslipidemia and the importance of providing educational intervention regarding diabetes self-care activities.

## Introduction

Dyslipidemia is the presence of high lipid levels in the blood, which is characterized by an increased level of total cholesterol (TC) (>5.2 mmol/L; 200 mg/dl), triglyceride (TG) (>1.7 mmol/L; 150 mg/dl), low-density lipoprotein-cholesterol (LDL-C) (> 3.4 mmol/L; 130 mg/dl) or decreased level of high-density lipoprotein-cholesterol (HDL-C) (<0.9 mmol/L; 35 mg/dl) [1-2]. An increase in the plasma concentration of lipids, such as cholesterol or triglycerides, is absorbed from the intestines and is carried throughout the body via lipoproteins for energy, steroid production, or bile acid formation. Major contributors to these pathways are cholesterol, low-density lipoprotein cholesterol (LDL-C), triglycerides, and high-density lipoprotein (HDL) [3-4]. An imbalance of any of these factors, either from organic or nonorganic causes, can lead to dyslipidemia [5].

The World Health Organization (WHO) reports that dyslipidemia is a major cause of ischemic heart disease (IHD) and cerebrovascular disease (CVD) [6]. In Saudi Arabia, dyslipidemia is a significant health problem and an important risk factor among type 2 diabetic patients [7-8]. Globally, half of type 2 diabetic patients have uncontrolled dyslipidemia and are more prone to cardiovascular disease [9]. Therefore, type 2 diabetic patients need to adopt a healthy lifestyle including a balanced diet and regular health checks [10-11]. A study done in China found that there is a low level of awareness among diabetic patients about the control and treatment of dyslipidemia. In addition, it was found that the independent risk factors of dyslipidemia were: advancing age, male, unhealthy lifestyles, positive family history of dyslipidemia, overweight, type 2 diabetes mellitus (DM), and hypertension [12].

A study done in Riyadh in 2019 reported that the prevalence of dyslipidemia ranged from 20% to 44%, and the highest rate was 44% for hypertriglyceridemia [7]. More than 25% of the participants had low levels of HDL-C, and one-fifth had high levels of total cholesterol. Males had a higher prevalence of all types of dyslipidemia (except LDL-C and total cholesterol). The majority of dyslipidemia types increase with advancing age. The education level was associated with dyslipidemia prevalence as it was more prevalent among illiterate patients compared to those having higher educational levels. Significant regional variations emerged from the data: the North region of Saudi Arabia was associated with the highest levels of triglycerides, while the Eastern region had the highest low levels of HDL-C [7].

A study conducted in Jeddah (a western Saudi city) showed that retired or unemployed patients had a higher prevalence of dyslipidemia [13]. Another survey in the southern region of Saudi Arabia showed dyslipidemia among males was higher than in females, which is a significant risk factor for causing low glycemic control, which leads to cardiovascular events [14]. The researcher attributed this to the lack of adherence to self-care activities among males compared to females as reported in previous literature [14].

Another study conducted in Almajmaah (central region of Saudi Arabia) showed dyslipidemia was more prevalent in patients with low levels of physical activity (63.4% of participants); moderate physical activity was observed in 33.4%, and only 3.1% were performing intense physical activity [14]. Another study done in Jeddah showed the prevalence of dyslipidemia was 78%. The prevalence of high total cholesterol was 38.7%, whereas high LDL was 43.5%, low HDL was 45.2%, and high triglycerides were 17.4%. Patients with type 2 diabetes had a nearly nine-fold higher risk to develop dyslipidemia compared with those who were without diabetes [15].

Patients 40 years and above were more likely to have dyslipidemia compared to the younger generation. Hypertensive patients were almost at an eight-fold higher risk of having dyslipidemia compared with those with normal blood pressure. Patients with diabetes were virtually at a tenfold risk to develop dyslipidemia compared with non-diabetics [16].

To our best knowledge, only a few studies have been conducted in Saudi Arabia to evaluate dyslipidemia control in type 2 diabetes. Therefore, in this study, we will assess the level of lipid parameters control in type 2 diabetic patients with dyslipidemia and its correlation with demographic factors and other comorbidities.

## Materials and methods

Research design

This was a cross-sectional study with a quantitative structured questionnaire.

Research setting

This study was conducted at the family medicine clinics at three of the Ministry of National Guard Health Affairs (MNGHA) primary health care centers. MNGHA has many primary health care centers distributed around different regions of Saudi Arabia. National Guard Comprehensive Specialized Center (NGCSC), King Abdul-Aziz City Housing (Iskan Yarmouk) Center, and Health Care Specialty Center (HCSC) are three of the main primary health care centers in Riyadh covering a large population with easy accessibility. Iskan Yarmouk clinics serve patients living in the military housing area in the eastern region of Riyadh while NGCSC serves patients living in the northern region while HCSC serves patients living in the eastern region of Riyadh. All centers provide general outpatient care with different specialties including family medicine clinics for chronic disease follow-up.

Sampling and sample size

The sample was recruited by simple random sampling from the list of patients who fit the inclusion and exclusion criteria that were provided by the data warehouse (best care). We collected the patients by random integer generator website [17].

Based on the data retrieved from the family medicine department office, it was found that the total population visiting the three centers was 24,0000 patients; about two-thirds were adults which equals 16,0000, and the last recorded rate of prevalence of diabetes mellitus type 2 in the three centers was 10% as per the 2019 annual report of the family medicine and primary health care department.

The sample size was calculated by OpenEPI (https://www.openepi.com) based on the data indicating that the frequency and prevalence of dyslipidemia among diabetes mellitus type 2 is 31% as per the study done at King Abdul-Aziz University in Jeddah [16].

The sample size calculated was 400 patients.

Inclusion and exclusion criteria

The inclusion criteria were being an adult over 35 years who visited the primary health care centers between 2019 and 2020 diagnosed as a type 2 DM patient, having available data about lipid profile and hemoglobin A1C, and having a medical record with more than 75% of data available. On the other hand, the exclusion criteria included being pregnant or the presence of familial hypercholesterolemia.

Data collection methods, instruments used, and measurements

The questionnaire was prepared with reference to previous studies in the literature. The content validity of the tool was ensured by consulting two experts both family medicine consultants. It was written in English. The completed questionnaire was checked and pretested for clarity and suitability in a small pilot study of 10 participants and then necessary corrections were made.

The questionnaire included two parts: Part 1 covered baseline sociodemographic and clinical data of the study participants (age, gender, comorbidities, occupation, educational level, smoking status, monthly income, diabetes duration, body mass index, frequency of eating fast food, frequency of eating vegetables and fruits, method of managing dyslipidemia, most common side effects of dyslipidemia drugs, ability to tolerate side effects, and frequency of forgetting to take medications). Part II recorded the laboratory measurements related to triglycerides, total cholesterol, low-density lipoprotein, high-density lipoprotein, and fasting blood sugar to hemoglobin A1C level. 

The data were retrieved from the medical database (best-care system), which is the electronic medical record of the hospital and all primary health care centers. Any missing data in the best care system was collected from the patient by telephone interview. For example, monthly income and educational level were mostly found to be missing in the medical records. Therefore, we had to make these phone interviews to collect the missing data.

According to National Cholestrol Education program (NCEP III), abnormal lipid level were defined as total cholesterol >240 mg/d, LDL> 160 mg/dL, HDL<40 mg/dL, TG >200 mg/dL [18].

## Results

A total of 418 participants were enrolled in the study. Table 1 shows the baseline demographic and clinical characteristics of the study participants. The mean age of the enrolled diabetic patients was (58.9±10.7) with no significant difference in the mean age of males and females. Females constituted 66.5% (n=275) of the enrolled diabetic patients, whereas males constituted around 34.2% (n=143). Around 60% (251) patients had hypertension and 63.9% (n=267) patients had obesity from the enrolled cohort of patients with T2DM.

Categorizing the participants based on their occupation showed that 59.1% (n=247) were unemployed, whereas 23.4% (n=98) were retired, 15.1% (n=63) were military employees, and 2.4% (n=10) were self-employed. Moreover, it was found that 39.7% (n=166) and 30.1% (n=126) were illiterate and had a primary school level education, respectively. However, 15.1% (n=63) had a high school level of education, 8.1% (n=34) had a secondary school level of education, and 6.9% (n=29) had a bachelor's degree or higher levels of education.

**Table 1:** Demographic and clinical characteristics of study participants

Variable	F(%)
Age (M±SD)	58.9±10.7
Gender	
1. Female	275 (65.8)
2. Male	143 (34.2)
Comorbidities	
1. Hypertension	251 (60)
2. Obesity	267 (63.9)
3. Hypothyroidism	62 (14.8)
4. Heart diseases	17 (4.1)
5. Retinopathy	83 (19.9)
6. Nephropathy	61 (14.6)
7. Neuropathy	74 (17.7)
Occupation	
1. Not employed	247 (59.1)
2. Self-employed	10 (2.4)
3. Military employed	63 (15.1)
4. Retired	98 (23.4)
Educational level	
1. Illiterate	166 (39.7)
2. Primary	126 (30.1)
3. Secondary	34 (8.1)
4. High school	63 (15.1)
5. Bachelor or higher	29 (6.9)
Smoking status	
1. Smoker	23 (5.5)
2. Non-smoker	395 (94.5)
Monthly income	
1. Less than 5000 SAR	207 (49.5)
2. 5000-10000 SAR	173 (41.4)
3. 10000-15000 SAR	24 (5.7)
4. More than 15 000	14 (3.3)
Diabetes duration	
1. Less than 5 years	55 (13.2)
2. 5-10 years	145 (34.7)
3. More than 10 years	218 (52.2)
Body mass index	
1. Underweight	1 (0.2)
2. Normal weight	29 (6.9)
3. Overweight	121 (28.9)
4. Obese	267 (63.9)

Majority of the study participants (94.5%, n=395) were non-smokers, whereas smokers constituted 5.5% (n=29). In addition, the results found that 49.5% (n=207) had a monthly income of less than 5000 SAR, whereas 41.4% (n=173) had a monthly income ranging between 5000 and 10000 SAR.

Exploring the duration of diabetes among the participants showed that 52.2% (n=218) had diabetes for more than 10 years, whereas 34.7% (n=145) and 13.2% (n=55) had diabetes for five to 10 years and less than five years, respectively.

Finally, it was found that 63.9% (n=267) were obese, and 28.9% (n=121) were overweight, whereas 6.9% (n=29) and 0.2% (n=1) were normal weight and underweight, respectively.

The results presented in Table 2 show the independent sample t-test to investigate the differences in dyslipidemia biomarkers between controlled and uncontrolled diabetic patients. The results revealed that there was a significant difference in triglycerides between controlled diabetic patients (1.5±0.74) and uncontrolled diabetic patients (1.8±1.01), p≤0.05. However, the results showed that low-density lipoprotein (LDL) levels did not differ significantly between controlled and uncontrolled diabetic patients. In addition, it was noticed that there is a significant difference in high-density lipoprotein (HDL) between controlled diabetic patients (1.25±0.68) and uncontrolled diabetic patients (1.1±0.26), p≤0.05. There was a significant difference in the levels of fasting blood sugars (FSB) between controlled diabetic patients (6.9±1.36) and uncontrolled diabetic patients (10.5±3.70), p≤0.05.

**Table 2:** independent sample t-test for the differences in dyslipidemia biomarkers (LDL: 130 mg/dL and above, TG: 150 mg/dL and above, TC: 200mg/dL and above, HDL less than 35mg/dL) between controlled and uncontrolled diabetes

Parameter	Controlled (HbA1c≤7%)	Uncontrolled (HbA1c˃7)	P-value
	(n=104)	(n=314)	
TG	1.5±0.74	1.8±1.01	≤0.05*
TC	4.5±0.99	4.6±1.10	0.41
LDL	2.9±0.99	2.9±0.96	1
HDL	1.25±0.68	1.1±0.26	≤0.05*
FSB	6.9±1.36	10.5±3.70	≤0.05*

The results described in Table 3 represent a chi-square analysis of the prevalence of dyslipidemia among type 2 diabetic patients. it was found that 82.1% (n=343) of the enrolled type 2 DM patients were dyslipidemic. A significant interaction was found between gender and dyslipidemia (Χ^2^=9.287, p=0.003). In addition, it was found that there is a significant interaction between HbA1c controlling and dyslipidemia (Χ^2^=7.584, p=0.005).

Moreover, it was found that there is a significant relationship between the educational level and dyslipidemia among the enrolled type 2 diabetic patients (Χ^2^=13.678, p=0.009). Finally, it was found that there was a significant interaction between the frequency of exercising and dyslipidemia (Χ^2^=18.452, p=0.0004).

**Table 3:** Prevalence of dyslipidemia among the study participants

Variable	Dyslipidemic (n=343)	Non-dyslipidemic	Χ^2^	P-value
		(n=75)		
Gender			9.287	0.003
1. Male	106 (30.9)	37 (49.3)		
2. Female	237 (69.1)	38 (50.7)		
Age			----	-----
1. Less than 40	12 (3.5)	0 (0)		
2. 40-50	68 (19.8)	15 (20)		
3. 51-60	123 (35.9)	21 (28)		
4. More than 60	140 (40.8)	39 (52)		
HbA1c			7.584	0.005
1. Controlled	76 (22.2)	28 (37.3)		
2. Uncontrolled	267 (77.8)	47 (62.7)		
Body mass index			-----	-----
1. Underweight	1 (0.29)	0 (0)		
2. Normal weight	20 (5.8)	9 (12)		
3. Overweight	97 (28.3)	24 (32)		
4. Obese	267 (63.9)	42 (56)		
Diabetes duration			0.752	0.687
1. Less than 5 years	43 (12.5)	12 (16)		
2. 5-10 years	121 (35.3)	24 (32)		
3. More than 10 years	179 (52.2)	39 (52)		
Educational level			13.678	0.009
1. Illiterate	145 (42.3)	21 (28)		
2. Primary	103 (30)	23 (30.7)		
3. Secondary	22 (6.4)	12 (16)		
4. High school	53 (15.5)	10 (13.3)		
5. Bachelor or higher	20 (5.8)	9 (12)		
Smoking			-----	------
1. Smoker	23 (6.7)	0 (0)		
2. Non-smoker	320 (93.3)	75 (100)		
Family history			5.678	0.0172
1. Yes	270 (78.7)	68 (90.7)		
2. No	73 (21.3)	7 (9.3)		
Frequency of eating vegetables and fruits			0.1316	0.7168
1. Less than 5 servings/day				
2. 5 or more servings/day	331 (96.5)	73 (21.3)		
	12 (3.5)	2 (2.7)		
Duration of physical activity			3.608	0.307
1. Less than 30 minutes	27 (7.9)	6 (8)		
2. 30-60 minutes	68 (19.8)	22 (29.3)		
3. More than 60 minutes	15 (4.4)	2 (2.7)		
4. Not practicing	233 (67.9)	45 (60)		
How often do you exercise?			18.452	0.0004
1. Less than 3 times per week	58 (16.9)	10 (13.3)		
2. 3-5 times per week	30 (8.7)	13 (17.3)		
3. More than 5 times per week	22 (6.4)	14 (18.7)		
4. Not practicing	233 (67.9)	38 (50.7)		
Are you physically active?			1.696	0.198
1. Yes	106 (30.9)	29 (38.7)		
2. No	237 (69.1)	46 (61.3)		

Table 4 shows the dyslipidemic patients' characteristics related to treatment behaviors and awareness. Regarding the frequency of eating fast food, only 8.2% always eat fast food, 22.7% sometimes, 19.5% usually, 18.7% rarely but 18.7% never eat fast food. Frequency of eating vegetables and fruits, 96.5% eat less than five servings/day and only 3.5% only eat five or more servings per day. Most side effects of dyslipidemia drugs were muscular pain in 4.9%, joint pain, and nausea or vomiting in 4.1%. Management of dyslipidemia was by medications in 68.5%, diet only in 1.5%, and diet and medications in 30%.

Patients forget to take medication frequently/or on a daily basis (18.1%), while "rarely" in 42.3% and "never" in 39.7% of participants. Around 80.2% were not aware of the side effects.

**Table 4:** Dyslipidemia patient’s characteristics related to treatment behavior and awareness

Variable	F(%)
Frequency of eating fast food	
1. Rarely	64 (18.7)
2. Never	75 (21.9)
3. Often	31 (9.0)
4. Sometimes	78 (22.7)
5. Usually	67 (19.5)
6. Always	28 (8.2)
Frequency of eating vegetables and fruits	
1. Less than 5 servings/day	331 (96.5)
2. 5 or more servings/ day	12 (3.5)
Management of dyslipidemia	
1. Medication	235 (68.5)
2. Diet	5 (1.5)
3. Both	103 (30)
Side effects related to dyslipidemia drugs	
1. Yes	41 (12)
2. No	302 (88%)
Most common side effects of dyslipidemia drugs	
1. Abdominal pain	7 (2.0)
2. Headache	1 (0.2)
3. Joint pain	14 (4.1)
4. Nausea or vomiting	14 (4.1)
5. Muscular pain	17(4.9)
6. Others	2 (0.5)
Are you able to tolerate side effects?	
1. Yes	34 (9.9)
2. No	34 (9.9)
3. I don’t know	275 (80.2)
How frequently do you forget to take your medication?	
1. Rarely	145 (42.3)
2. Daily	11 (3.2)
3. Never	136 (39.7)
4. Frequently	51 (14.9)

## Discussion

The study aimed to assess dyslipidemia and control in type 2 diabetic patients, retrieve the level of lipid parameters control in diabetic patients with dyslipidemia, retrieve diabetes control through HbA1c level, and correlate dyslipidemia with diabetic control and other patient characteristics.

A study by Reaven [19] found that triglyceride level is not elevated along with the degree of hyperglycemia directly, but correlated with insulin resistance. TG consists of three fatty acid molecules. Free fatty acids (FFA) play a crucial role in TG production in the liver and partly in the intestine. Hypertriglyceridemia is a result of abnormalities in glucose metabolism. In patients with impaired glucose tolerance, the basic defect is postulated to be the loss of normal insulin sensitivity, leading to compensatory hyperinsulinemia and increased VLDL-TG secretion. Patients with type 2 diabetes have relative insulin deficiency, and the elevated FFA levels increase hepatic VLDL-TG secretion.

The results of our study have revealed that there was a significant difference in triglycerides between controlled diabetic patients (1.5±0.74) and uncontrolled diabetic patients (1.8±1.01), p≤0.05. In addition, it was found that there are significant differences in HDL between controlled diabetic patients (1.25±0.68) and uncontrolled diabetic patients (1.1±0.26), p≤0.05. Moreover, it was found that there was a significant difference in the levels of fasting blood sugars (FSB) between controlled diabetic patients (6.9±1.36) and uncontrolled diabetic patients (10.5±3.70), p≤0.05. These findings are consistent with the findings reported by Liu et al. [12] who found that there is a significant difference in HDL, TG, and FSB between controlled and uncontrolled diabetic patients. However, the study found no significant difference in LDL levels between controlled and uncontrolled diabetic patients, which could be referred to the use of medications such as metformin, which has been reported to significantly cause a reduction in LDL levels even in uncontrolled diabetic patients. These results are consistent with the findings reported by Singh et al. who found no significant difference in LDL levels between controlled and uncontrolled diabetic patients [20]. Moreover, the findings of the present study are in line with the findings of the cross-sectional comparative cohort study conducted at Creek General Hospital in Pakistan by Kidwai et al. [21], on 150 patients with diabetes mellitus were randomly selected, as there was a significantly high level of triglycerides in uncontrolled diabetes, p≤0.002. Another retrospective cross-sectional study accomplished at the King Abdul-Aziz University Hospital (KAUH) in Jeddah, on 206 participants, found that patients with HbA1c levels above 7% (poor glycemic control) had significantly higher TG levels(r=0.16, p=0.02) compared to the group with HbA1c <7% (good glycemic control); however, no significant difference was found in the other parameters(HDL-C) or (FBS) p= 0.20, p= 0.32, respectively [13]. Another study reported similar results displaying significantly elevated levels of TC, LDL-C, and TGs along with reduced HDL-C in subjects with HbA1c over 7% compared to those subjects with HbA1c <7%. It seems that patients with good glycemic control have less dyslipidemia compared to patients with poor glycemic control. Hussain et al. [22] also reported that HbA1c could be a predictor of TG levels.

In our study, a significant interaction was found between gender and dyslipidemia (Χ^2^=9.287, p=0.003). This might be referred to as sex-related differences, especially hormonal differences between males and females. Our findings are consistent with the findings reported by Soriano-Maldonado et al. who provided research-based evidence that dyslipidemia is heterogeneous based on gender and it differs significantly between males and females in terms of prevalence and risks [23].

In addition, it was found that there is a significant interaction between HbA1c control and dyslipidemia (Χ^2^=7.584, p=0.005). A cross-sectional comparative cohort study revealed that LDL-C and TG's had a strong correlation (p< 0.05) with each other as well as with increasing HbA1c whereas an inverse relationship is seen between HbA1c and HDL [24].

Another cross-sectional study conducted on 275 volunteered diabetic patients showed significant increase in all examined lipid parameters (TC, TG, LDL-C) and calculated indexes (lipid accumulation product, visceral adiposity index) across HBA1c three groups, with exception of HDL-C that decreased along with increment in HBA1c, TC (OR = 1.46, 95% CI (1.18-1.81), P = 0.001), TG (OR = 1.44, 95% CI (1.18-1.76), P < 0.001), and LDL-C (OR = 1.42, 95% CI (1.11-1.83), P = 0.006), increased by 1 mmol/L each, probabilities of higher HBA1c increased by 46%, 44% and 42%, respectively [23]. High TC and TG were the independent predictors of higher HBA1c. On the other hand, an increase in HDL-C (OR = 0.47, 95% CI (0.23-0.98), P = 0.043), by 1 mmol/L reduced the probability of higher HBA1c by 53%. Correlation of HBA1c with TC and TG, but not with HDL-C and LDL-C was shown by previous studies. Whereas, on the contrary, Babikr et al. reported an association of HBA1c with HDL-C and LDL-C, but not with TC and TG [25]. On the other hand, a correlation has been reported by several other studies [26-27] that found a positive relationship between HbA1c and high TG levels.

A cross-sectional study was conducted on Afghan patients with type 2 DM to assess the relationship between glycemic control (HbA1c) and serum lipid profile as well as to evaluate the importance of HbA1c as an indicator of dyslipidemia in patients with type 2 DM in an Afghan population, the study showed that correlation between HbA1c and HDL-C was negative, however, there was a positive, significant correlation between HbA1c and TC, LDL-C, and TGs, and high levels of FBS, HbA1c, TC, HDL-C and LDL-C in females compared with male patients, this may be attributed to the effects of sex hormones on body fat distribution, which leads to differences in changed lipoproteins [22].

A study carried out at Ayub Teaching Hospital Abbottabad revealed that female diabetic patients have an increased frequency of low levels of serum HDL as compared to males [28]. Another study revealed that females had significantly higher values for TC (p<0.001), HDL-C (p=0.002), LDL-C (p<0.001), and HbA1c (p=0.009) compared to the males [28]. On the other hand, a cross-sectional cohort study showed that differences in lipid levels between women and men with type 2 DM change substantially across the life span. For patients not treated with a statin, women had lower LDL-C levels than men before the age of 45 years and higher LDL-C levels after the age of 50 years. Statin treatment lowered LDL-C levels in both women and men, but women still had higher LDL-C levels than men after the age of 55 years. HDL-C levels were consistently higher in women than in men in all age groups, regardless of statin treatment. TG levels were higher in men than in women before the age of 60 years, regardless of statin treatment [29]. Furthermore multilevel logistic regression analysis (clinical data extracted from electronic medical records) revealed that women had significantly higher mean total cholesterol (TC), LDL-C, and HDL-C serum levels compared to men (P < 0.0001 for all comparisons). Serum triglycerides levels were not statistically different between groups. Conversely, LDL/HDL ratio was slightly, but significantly, higher in type 2 DM men than in women (P < 0.0001) [30].

Furthermore, our findings revealed that a number of dyslipidemic patients used diet as a management approach to dyslipidemia while two-thirds of the patients were on medications to manage dyslipidemia, which might be recommended in the advanced stages of diabetes whereas diet is considered a self-care activity more than a management approach. This is evidenced by the findings reported by Jialal and Singh who reported that medications, especially statin therapy, is the therapy of choice in dyslipidemic patients [31].

Around 82% of patients do not forget medication which shows good compliance to medication. 

Despite the significant findings reported in the present study, still, several limitations are preventing the generalization of the study findings. These limitations include the psychometric properties of the data collection tool used for completeness of the missing data in the patients' medical records. In addition, the validity and reliability of the data obtained via phone interviews were retrieved from patients, not from medical records. In addition, there were geographical limitations; this study was performed in Riyadh city and did not include type 2 diabetic patients from other geographical zones, which might affect the findings of the study due to the lack of variations in socioeconomic determinants. Further, this is a retrospective study that used available type 2 DM patients' data.

## Conclusions

The study concluded that significant interactions were found between gender and dyslipidemia, HbA1c control, educational level and frequency of exercising and dyslipidemia among the enrolled type 2 DM patients. Based on the findings of our study, it is recommended to conduct intensive educational and awareness campaigns to increase diabetic patients’ awareness regarding diabetes self-care practices and management of diabetic complications. 

## Appendices

Table 5 shows the questionnaire distributed to patients for this study. 

**Table 5:** Questionnaire

Variable	
patient number file	
Age	
Gender	
1. Female	
2. Male	
Comorbidities	
1. Hypertension	
2. Obesity	
3. Hypothyroidism	
4. Heart diseases	
5. Retinopathy	
6. Nephropathy	
7. Neuropathy	
Occupation	
1. Not employed	
2. Self-employed	
3. Military employed	
4. Retired	
Educational level	
1. Illiterate	
2. Primary	
3. Secondary	
4. High school	
5. Bachelor or higher	
Smoking status	
1. Smoker	
2. Non-smoker	
Monthly income	
1. Less than 5000 SAR	
2. 5000-10000 SAR	
3. 10000-15000 SAR	
4. More than 15 000	
Diabetes duration	
1. Less than 5 years	
2. 5-10 years	
3. More than 10 years	
Body mass index	
1. Underweight	
2. Normal weight	
3. Overweight	
4. Obese	
Family history	
1. Yes	
2. No	
Frequency of eating vegetables and fruits	
1. Less than 5 servings/day	
2. 5 or more servings/day	
Are you physically active	
1. Yes	
2. No	
Duration of physical activity	
1. Less than 30 minutes	
2. 30-60 minutes	
3. More than 60 minutes	
4. Not practicing	
How often do you exercise?	
1. Less than 3 times per week	
2. 3 -5 times per week	
3. More than 5 times per week	
4. Not practicing	
Frequency of eating fast food	
1. Rarely	
2. Never	
3. Often	
4. Sometimes	
5. Usually	
6. Always	
Management of dyslipidemia	
1. Medication	
2. Diet	
3. Both	
Side effects related to dyslipidemia drugs	
1. Yes	
2. No	
Most common side effects of dyslipidemia drugs	
1. Abdominal pain	
2. Headache	
3. Joint pain	
4. Nausea or vomiting	
5. Muscular pain	
6. Others	
Are you able to tolerate side effects?	
1. Yes	
2. No	
3. I don’t know	
How frequently do you forget to take your medication?	
1. Rarely	
2. Daily	
3. Never	
4. Frequently	
